# Open questions on the structures of crystalline water ices

**DOI:** 10.1038/s42004-020-00349-2

**Published:** 2020-08-07

**Authors:** Thomas Loerting, Violeta Fuentes-Landete, Christina M. Tonauer, Tobias M. Gasser

**Affiliations:** 1 Institute of Physical Chemistry, University of Innsbruck, A-6020 Innsbruck, Austria; 2 Max-Planck-Institut für Chemische Energiekonversion, D-45470 Mülheim an der Ruhr, Germany

**Keywords:** Physical chemistry, Condensed-matter physics, Solid-state chemistry

## Abstract

Water can form a vast number of topological frameworks owing to its hydrogen-bonding ability, with 19 different forms of ice experimentally confirmed at present. Here, the authors comment on open questions and possible future discoveries, covering negative to ultrahigh pressures.

The hydrogen bond as envisioned by Linus Pauling is key to the structures of H_2_O ices. A hydrogen bond can be characterised as O–H⋯O, where the hydrogen atom is linked via a short covalent bond to the donor oxygen and via the longer bond to the acceptor. The one feature that makes ice structures so diverse is that four hydrogen bonds can be formed by a water molecule, although it has only three atoms. Thereby, two atoms may act as H donors, two as H acceptors. In ice, as opposed to water clusters or liquid water, all four hydrogen bonds are developed, resulting in a tetrahedral coordination around each water molecule. This local coordination motif is called the Walrafen pentamer and is found in almost all ice structures^1^.

## Hydrogen bonding at ultrahigh pressure

**Fig. 1:**
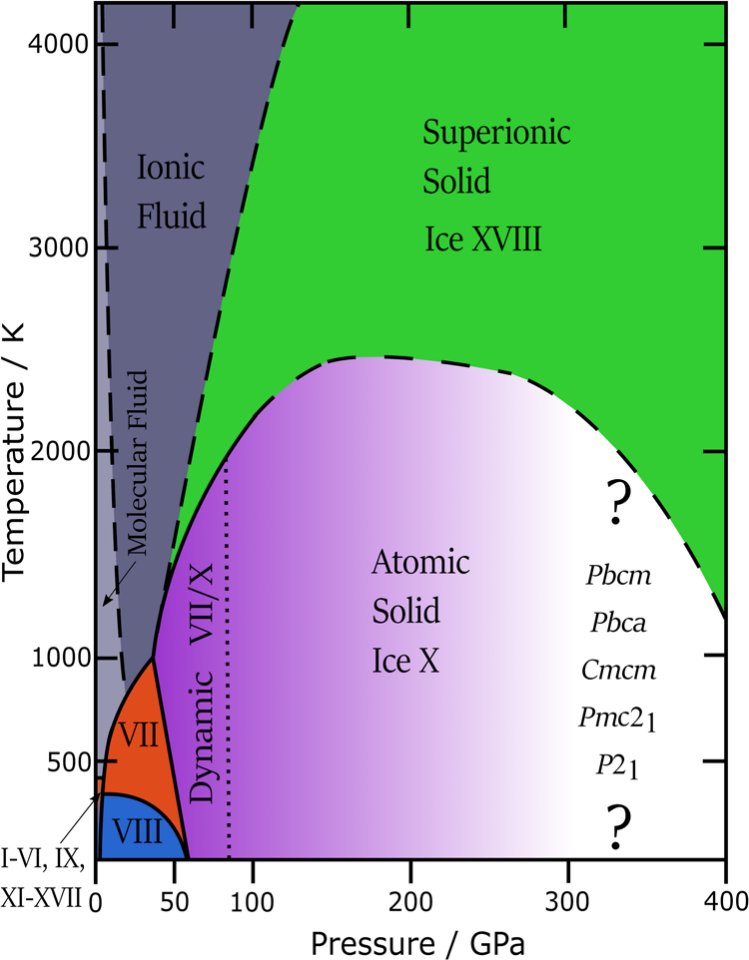
Phase diagram at high to ultrahigh pressure. Stable phases of ice and liquid water at temperatures up to 4000 K and to ultrahigh pressures of 400 GPa. Grey indicates molecular and ionic liquid water. Blue indicates H-ordered ice VIII, red indicates H-disordered ice VII. Purple indicates the atomic solid ice X, in which the H atoms are at the centre between two O atoms. The dotted line separates ice X from ice VII, in which the H atoms are centred in a time-average, but localised in a snapshot. Green indicates ice XVIII, in which the H atoms are mobile and delocalised in a fixed lattice of O atoms. Dashed lines are estimates. Predicted post ice X polymorphs as candidates for future experimental discovery are listed at the ultrahigh pressure end.

Figure 1 summarises the phase diagram for water in a very broad pressure regime, up to pressures as high as encountered in the interior of the ice giants, Uranus and Neptune. Only at ultrahigh pressures, exceeding 50 GPa, the Walrafen pentamer breaks down. In this pressure regime, the hydrogen atom becomes centred between the two oxygen atoms. The molecular nature of H_2_O breaks down in two stages. First, the H atoms jump back and forth between the two O atoms (labelled dynamic VII/X in Fig. 1). Second, the H atoms remain static in the centre (labelled atomic solid ice X). Roman numerals are used for ice phases, following the chronological order of the deduction of their crystal structures. Ice X was made in the laboratory in 1984^2^ and represents the 10^th^ polymorph of H_2_O. It does not melt when it is heated beyond 2500 K. It experiences a dynamic transition instead: the H atoms start to be mobile in a lattice of O atoms remaining at fixed positions—a property known as superionicity. This ice was discovered only very recently through X-ray experiments on laser-shocked water, and is called ice XVIII since 2019^3^. Ice XVIII is almost as conductive as metals are. In fact, delocalised H atoms in superionic ice take the same charge carrier role as delocalised electrons in metals. Based on the conditions of the experiments, Uranus and Neptune might actually contain superionic ice within their mantles, but this is uncertain since it is unclear how contaminations, e.g., from carbon, affect their behaviour. How the ice structure is affected at even higher pressures can be only predicted in simulations. Shock-wave experiments and bevelled diamond-anvil cells are limited to 300 GPa (3 Mbar), currently. Above about 300 GPa, a range of many other post ice X structures is predicted that have yet to be discovered in experiment. Their space groups are given at the high-pressure end in Fig. 1^4,5^.

## Hydrogen bonding at negative pressure

**Fig. 2:**
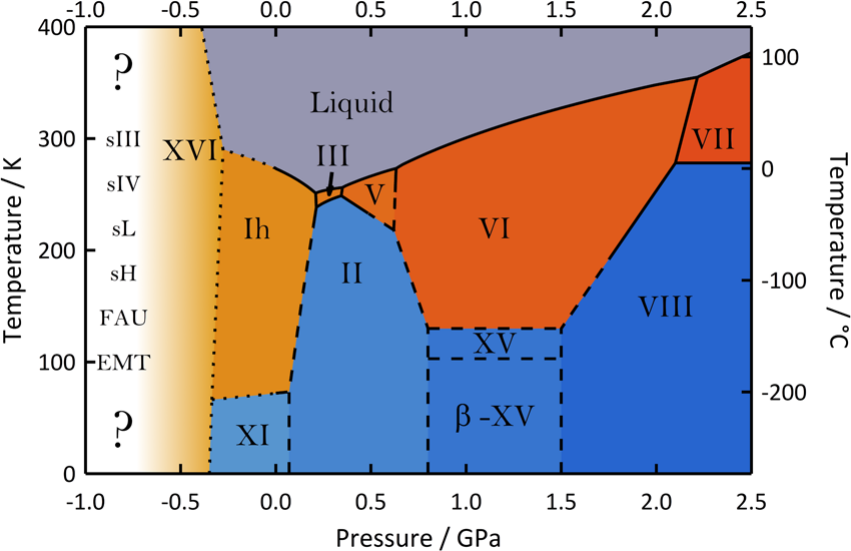
Phase diagram at negative to intermediate pressure. Stable phases of ice and liquid water up to 2.5 GPa and 400 K, excluding water vapour and metastable phases. Dashed lines indicate extrapolations based on experiments at higher temperature. Dotted lines at negative pressure are sketched based on simulations by Conde et al. in ref. ^9^ and shifted to match experimental data at positive pressure. Grey indicates liquid water. Blue indicates H-ordered ices, orange/red indicates H-disordered ices. Shades of colour indicate density. Predicted low-density ice polymorphs as candidates for future experimental discovery derived from clathrate hydrate and zeolite structures are listed at the far negative pressure end.

Turning to the negative pressure regime, ultralow-density forms of ice are encountered that are significantly less dense than common hexagonal ice found on Earth’s surface. These are open structures that have cages large enough to fit even guest molecules. Such structures may be derived from naturally occurring clathrate hydrates, such as methane hydrates. Without guest molecules, such open ice forms are supposed to form from hexagonal ice under tensile stress. Such experiments have not been successfully done, though, mainly because icicles rupture easily when pulling on them. By contrast, experiments on liquid water at negative pressure were done in the past, avoiding cavitation and the transition to the stable vapour^6^. Nonetheless, two empty clathrate hydrates could be realised in experiments that are now known as ices XVI^7^ and XVII^8^. Ice XVI is prepared in vacuo by pumping on a neon-filled clathrate hydrate of cubic structure II over extended periods, thereby emptying it^7^. A similar strategy was adopted to produce ice XVII starting from hydrogen-filled ice as a precursor^8^. On thermodynamic grounds, one would expect them to be stable only at negative pressures (see predicted stability range of ice XVI in Fig. 2), but to revert to ice I at ambient pressure. This is indeed the case, but below 130 K they are kinetically stable. It seems likely that ices XVI and XVII will not remain the only known ultralow-density ices. Computer simulations have identified a number of structures stable at negative pressure^9,10^. These are derived from known guest-filled clathrate hydrates or zeolite type networks (listed at the left in Fig. 2). Empty hydrogen-bond networks related to these mother phases have yet to be prepared in experiments. They might be produced in future research either as metastable ices in vacuo by emptying the mother clathrate hydrates, by freezing water under tensile stress or by pulling on icicles.

## The versatile intermediate pressure

Apart from the ultralow- and ultrahigh-density ices described so far, all other experimental discoveries pertain to the highly versatile intermediate-pressure range, between about 0.1 and 2.5 GPa as depicted in Fig. 2. In total, 13 out of the currently 19 known ice polymorphs are found in this relatively narrow pressure range. Ices II–IX were discovered prior to the 1970s. Ices XII–XV and β–XV were made in the last two decades. Ices VI and VII in fact exist in the interior of Earth and return to the surface in the form of diamond inclusions^11^. They have, therefore, been declared as a mineral by the international mineralogic association. At depths of several hundred kilometres, these ices might be of relevance to build friction between cold-subduction slabs, resulting in seismic events one could term “ice quakes.” High-pressure forms of ice are even more abundant in space, where the icy moons are covered in layers of ice up to 1000 km thick so that their own weight suffices to transform the ices to high-pressure polymorphs such as ice III, V, VI, or VII. On Earth pressures within even the deepest glaciers are not high enough to transform hexagonal ice to high-pressure ice forms. The density increase from ice I to ice III to ice V to ice VI and to ice VII (indicated in Fig. 2 through shades of orange/red) is accommodated in the O-atom network in different ways: first, the hydrogen bonds are compressed and bent in ice III. Then the topology and ring sizes change—rather than the six-membered rings in hexagonal ice, ice V contains both smaller and larger rings. Also, ring threading allows for higher densities, as is the case for ice IV. In ices VI and VII, the high densities are reached through two independent H_2_O networks that are fully interpenetrating.

## Geometrically frustrated ices

The single property that has resulted in the most discoveries of ice phases is hydrogen order. In an ice crystal, both the H atoms and O atoms may occupy lattice positions, resulting in oriented water dipoles (coloured in blue in Fig. 2). Such ices have very low static dielectric constants of about 4. However, in most cases, especially when crystallising the ice from liquid water, only the O atoms are ordered, but not the H atoms (coloured in orange/red)—a property known as geometrical frustration. The water molecules still obey the Bernal–Fowler ice rules^12^. However, the water dipoles are oriented randomly causing dielectric constants of above 100. Currently, the six hydrogen disorder-order pairs I_h_–XI, III–IX, V–XIII, VI–XV, VII–VIII, and XII–XIV are known. The order-disorder transition temperature varies between 72 K for the ice I_h_/XI couple and 273 K for the ice VII/VIII couple. To actually make H-ordered ices and to release the frustration in the H-subnetwork kinetics is the key challenge. It has taken until the 1970s before Kawada achieved to make partly H-ordered ice XI from its disordered parent ice I_h_. The ordering process requires waiting times of days at <72 K^13^, but only if extrinsic defects are injected into the hydrogen-bond network through dopants. HCl and KOH have been the most successful dopants, where KOH allows accessing ice XI, and HCl allows accessing the H-ordered high-pressure ices XIII–XV^14^. As of now, it is still unknown why HCl accelerates ordering dynamics in high-pressure ices, and KOH in low-pressure ice, but not the other way around.

One specific and unique case that has received much attention lately is ice VI. Not only one, but in fact 45 different types of H order can be imagined that all obey the Bernal–Fowler ice rules. The antiferroelectric ice XV structure discovered in 2009 is only one of the H-ordered pendants of disordered ice VI^15^. In our experiments we recently discovered a second one, which we call ice β–XV^16^. This represents the 19^th^ polymorph of ice, where elucidation of its crystal structure based on diffraction experiments is ongoing work. While still being contested, this makes the case for the first phase of ice, in which transitions between two differently ordered H-sublattices may be studied—and it is fully open how such a transition progresses. Eighty-five years ago, Pauling considered all H-ordered structures as degenerate, which can be accessed through permutation of H atoms. It is clear now, though, that there are subtle differences in enthalpy, lattice parameters and density. Yet, it is still unclear for all ices known today whether a more stable arrangement of the H atoms exists, and whether this will be realised experimentally someday.

## Outlook

Even at ambient pressure ice still holds surprises, as shown through the realisation of a cubic stacking sequence in ice I without hexagonal stacking faults in 2020^17,18^. That is, it is still far from clear how many condensed phases of water can be distinguished. A recent machine learning study on ice phases actually suggests in total 51 ice polymorphs, more than 30 of which have not yet been reported in experiments^19^. If the stable structures that are predicted in simulations can actually be achieved in experiments, we expect to discover more ice phases in the future than have been discovered up to now. Rather than the candidates indicated between the question marks in Figs. 1 and 2, we think that most likely the next ice to be realised in experiments will be in the intermediate pressure range, e.g., the H-disordered pendant to ice II or the H-ordered pendant to ice IV. These are signs for a field that has not reached maturity yet! That is, in spite of a more than hundred year old history of research on ice structures, the field is still young and evolving more than ever.

## References

[1] Walrafen GE (1964). Raman spectral studies of water structure. J. Chem. Phys..

[2] Hirsch KR, Holzapfel WB (1984). Symmetric hydrogen bonds in Ice X. Phys. Lett. A.

[3] Millot M (2019). Nanosecond X-ray diffraction of shock-compressed superionic water ice. Nature.

[4] Militzer B, Wilson HF (2010). New phases of water ice predicted at megabar pressures. Phys. Rev. Lett..

[5] Hermann A, Ashcroft NW, Hoffmann R (2012). High pressure ices. Proc. Natl. Acad. Sci. USA.

[6] Herbert E, Caupin F (2005). The limit of metastability of water under tension: theories and experiments. J. Phys.: Condens. Matter.

[7] Falenty A, Hansen TC, Kuhs WF (2014). Formation and properties of ice XVI obtained by emptying a type sII clathrate hydrate. Nature.

[8] del Rosso L, Celli M, Ulivi L (2016). New porous water ice metastable at atmospheric pressure obtained by emptying a hydrogen-filled ice. Nat. Commun..

[9] Conde MM, Vega C, Tribello GA, Slater B (2009). The phase diagram of water at negative pressures: Virtual ices. J. Chem. Phys..

[10] Liu Y (2019). An ultralow-density porous ice with the largest internal cavity identified in the water phase diagram. Proc. Natl. Acad. Sci..

[11] Tschauner O (2018). Ice-VII inclusions in diamonds: Evidence for aqueous fluid in Earth’s deep mantle. Science.

[12] Bernal JD, Fowler RH (1933). A theory of water and ionic solutions, with particular reference to hydrogen and hydroxyl ions. J. Chem. Phys..

[13] Kawada S (1972). Dielectric dispersion and phase transition of KOH doped ice. J. Phys. Soc. Jpn..

[14] Salzmann CG, Radaelli PG, Slater B, Finney JL (2011). The polymorphism of ice: five unresolved questions. Phys. Chem. Chem. Phys..

[15] Salzmann CG, Radaelli PG, Mayer E, Finney JL (2009). Ice XV: a new thermodynamically stable phase of ice. Phys. Rev. Lett..

[16] Gasser TM (2018). Experiments indicating a second hydrogen ordered phase of ice VI. Chem. Sci..

[17] Komatsu K (2020). Ice Ic without stacking disorder by evacuating hydrogen from hydrogen hydrate. Nat. Commun..

[18] del Rosso L (2020). Cubic ice Ic without stacking defects obtained from ice XVII. Nat. Mater..

[19] Engel EA, Anelli A, Ceriotti M, Pickard CJ, Needs RJ (2018). Mapping uncharted territory in ice from zeolite networks to ice structures. Nat. Commun..

